# New developments in epidemiological research on dietary patterns associated with chronic kidney disease

**DOI:** 10.3389/fnut.2025.1665833

**Published:** 2026-01-12

**Authors:** Huixing Huang, Cuiying Yu, Pengjie Sha, Leilei Chen, Rongrong Wang, Yanli Wei

**Affiliations:** 1 The Second Clinical College of Guangzhou University of Chinese Medicine, Guangzhou, China; 2 School of Nursing, Guangzhou University of Chinese Medicine, Guangzhou, China; 3 The Second Affiliated Hospital of Guangzhou University of Chinese Medicine, Guangzhou, China

**Keywords:** chronic kidney disease, clinic trials, diet, dietary pattern, epidemiological research

## Abstract

Chronic kidney disease (CKD) represents a global health challenge, for which dietary modification serves as a profound preventive and interventional strategy. This article systematically examines and compares evidence from experimental and observational studies regarding the associations between the Mediterranean Diet (MD), Dietary Approaches to Stop Hypertension (DASH), New Nordic Renal Diet (NNRD), lacto-ovo-vegetarian diet (VD), and Chinese dietary patterns with CKD. Comprehensive analysis indicates that both MD and DASH exert significant preventive and protective effects against CKD. The NNRD demonstrates unique advantages in the precise management of phosphorus and protein intake among mid-to-late stage CKD patients. Meanwhile, VD, which is predominantly based on plant protein, contributes to the improvement of renal function parameters. However, in contrast, this review identifies a critical gap in the current evidence base: a severe scarcity of high-quality studies focusing on traditional Chinese dietary patterns. In conclusion, although international dietary patterns offer valuable insights, future research should prioritize the investigation of local dietary habits. Large-scale prospective studies are warranted to develop and validate dietary patterns tailored for CKD prevention and management within the Chinese population, thereby providing more targeted dietary recommendations.

## 1 Introduction

Chronic kidney disease (CKD), characterized by the gradual loss of kidney function, spans from mild renal impairment to severe renal failure. It has become a global health concern (1), affecting approximately 10% of the world’s population (2). In China, according to the analysis of the sixth national survey on chronic diseases and their risk factors, the prevalence of CKD is approximately 8.2% (3). As the number of patients continues to increase and the economic burden grows heavier, it is urgent to adopt effective primary prevention strategies to reduce the escalating disease burden.

The essence of primary prevention for CKD involves modifying risk factors (4). This includes managing hypertension, hyperglycemia, hyperlipidemia, obesity, and cardiovascular diseases to achieve the desired effect of preventing CKD. Dietary adjustments are particularly significant modifiable risk factors in preventing the progression of CKD (5, 6). In recent years, there has been widespread scholarly attention on adopting scientific dietary approaches to prevent CKD. Compared to focusing on individual dietary components, dietary patterns place greater emphasis on the synergistic and/or antagonistic interactions among nutrients, which facilitates people’s understanding and adoption of relevant recommendations (7). Currently, both the Mediterranean Diet (MD) and the Dietary Approaches to Stop Hypertension (DASH) have been confirmed by numerous studies as dietary patterns that positively impact health (8). However, both MD and DASH are rooted in Western dietary culture, and their food compositions differ significantly from the traditional dietary habits of Chinese residents. Whether these internationally validated dietary patterns are equally applicable to the Chinese population and can be sustainably adopted still requires further localized research evidence.

Therefore, this article systematically reviews evidence pertaining to the associations of MD, DASH, NNRD, VD, and Chinese dietary patterns with CKD, aiming to conduct a systematic comparison and synthesis of dietary pattern studies across different cultural contexts. This will help assess the generalizability of international evidence and the potential of local patterns, thereby providing a reference for exploring dietary strategies suitable for CKD prevention and management in the Chinese population.

## 2 Materials and methods

This study was conducted in accordance with the Preferred Reporting Items for Systematic Reviews and Meta-Analyses (PRISMA) guidelines (9), aiming to systematically evaluate the progress in epidemiological research on the associations between different dietary patterns and chronic kidney disease CKD.

### 2.1 Inclusion and exclusion criteria

#### 2.1.1 Inclusion criteria

(1)    Participants: Adults aged ≥ 18 years, including individuals with clinically diagnosed CKD, those at risk of developing CKD, or individuals presenting with markers of renal impairment (such as estimated glomerular filtration rate (eGFR), urinary phosphorus).
(2)    Intervention/Exposure: Clearly defined dietary patterns, including the MD, DASH, NNRD, VD, and Chinese dietary patterns.
(3)    Comparator: Usual diet or other dietary patterns.
(4)    Outcomes: Primary outcomes were the incidence and progression rate of CKD (e.g., decline in eGFR). Secondary outcomes included changes in biomarkers such as urinary protein and urinary phosphorus.
(5)    Study Types: Experimental studies and observational studies (cohort studies, cross-sectional studies).

#### 2.1.2 Exclusion criteria

(1)    Non-original research (e.g., reviews, commentaries, case reports, conference abstracts, and study protocols without reported results);
(2)    Studies focusing solely on single nutrient supplementation rather than overall dietary patterns;
(3)    Participants with end-stage renal disease (ESRD) who were receiving renal replacement therapy;
(4)    Non-human studies;
(5)    Full text unavailable or insufficient data extractable from the full text.

#### 2.1.3 Operational definition of dietary patterns

To ensure consistency and reproducibility in the literature screening process, the following operational criteria were used to classify dietary patterns. A study was included in a specific category if it met any of the following conditions:

(1)    MD: The study used a recognized MD score (e.g., MedDiet Score, aMED) or explicitly described the dietary intervention/intake as adhering to the core principles of MD.
(2)    DASH: The study applied a established DASH adherence score or explicitly stated that the dietary intervention was designed to implement DASH.
(3)    NNRD: The study explicitly named the intervention as the “New Nordic Renal Diet” or described it as following the established NNRD protocol.
(4)    VD: The study explicitly defined the diet as “lacto-ovo-vegetarian,” specifying the inclusion of dairy products and eggs and the exclusion of meat, poultry, and fish.
(5)    Chinese Dietary Pattern: The study identified a dietary pattern derived from dietary data in a Chinese population using analytical methods (e.g., factor or cluster analysis) and provided a specific name and description for the pattern. The nomenclature and definition provided by the original authors were adopted.

### 2.2 Literature search

**FIGURE 1:**
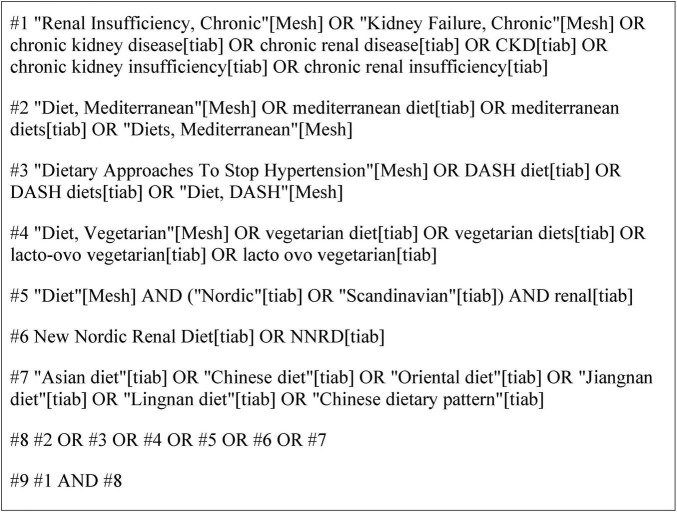
PubMed search strategy.

The search for literature was carried out until 30 May, 2025 on the following electronic databases: (1) MEDLINE (PubMed); (2) Embase; (3) Cochrane Library and (4) Web of Science. The main search string included the following words: (Renal Insufficiency, Chronic OR Kidney Failure, Chronic OR Chronic Kidney Disease OR Chronic Renal Disease OR CKD) AND (Diet, Mediterranean OR Mediterranean Diet OR Diets, Mediterranean OR Dietary Approaches To Stop Hypertension OR DASH Diet OR DASH Diets OR Diet, Vegetarian OR Vegetarian Diet OR Lacto-Ovo Vegetarian OR New Nordic Renal Diet OR NNRD OR Chinese Diet OR Asian Diet OR Jiangnan Diet OR Lingnan Diet OR Oriental Diet). An example of the complete search strategy (for PubMed) is provided in Figure 1.

Additionally, to minimize the risk of omission, the reference lists of all included studies and relevant reviews were manually screened. No restrictions were imposed on publication date or publication status. The search was limited to articles published in English or Chinese.

### 2.3 Data collection

The results obtained from the electronic search were independently evaluated by two reviewers (HX and CY). Initially, duplicate records were automatically removed using reference management software. Subsequently, the two reviewers independently conducted a preliminary screening of the remaining records based on titles and abstracts to identify potentially eligible studies. For the records retained after preliminary screening, the same two reviewers retrieved and thoroughly reviewed the full texts to determine final eligibility according to predefined inclusion and exclusion criteria. Any disagreements arising during the literature screening or data extraction processes were initially resolved through discussion and consensus between the two reviewers (HX and CY). If a consensus could not be reached, the disagreement was referred to a third reviewer (PJ) for arbitration, whose decision was considered final. Four reviewers (HX, CY, PJ, and LL) extracted the following information from each study: first author, year of publication, country, study design, sample size, participant characteristics (e.g., age, CKD stage), dietary pattern, intervention duration or follow-up time, and key CKD-related outcome measures Table 1.

**TABLE 1:** Sample and intervention characteristics of the studies.

References	Country	Study design	Sample	Participant characteristics	Dietary pattern (assessment method)	Intervention/ follow-up	Key CKD-related outcomes
Khatri et al. (16)	USA (NY)	PC	900	64 (59–71) y; no CKD	MD (MeDi score, 0–9)	Median 6.9 y	Each 1-point ↑ in MeDi score linked to 17%↓ odds of eGFR < 60 (OR 0.83); score ≥ 5 vs. <5 linked to 50% ↓ risk.
Asghari et al. (17)	Iran (Tehran)	PC	1212	43.5 ± 13.5 y; no CKD	MD (tMDS, 0–8)	Median 6.1 y	Highest tMDS quartile vs. lowest linked to 51% ↓ CKD risk (HR 0.49).
Hu et al. (22)	USA	PC	2403	21–74 y; CKD 2–4	Alternate MD (aMed score)	14 y	Highest aMed adherence vs. lowest linked to 25% ↓ CKD progression risk (HR 0.75).
Asghari et al. (28)	Iran (Tehran)	PC	1630	42.8 ± 14.2 y; no CKD	DASH (DASH score based on 168-item FFQ)	Median 6.1 y	Highest DASH quintile vs. lowest linked to 59% ↓ CKD odds (OR 0.41, 95% CI: 0.24–0.70).
Banerjee et al. (30)	USA	PC	1110	≥18 y; CKD 3; hypertension	DASH (24-h dietary recall)	Median 7.8 y	ESRD incidence: 24.5% (Q1) vs. 15.9% (Q5); Q1 vs. Q5 had higher ESRD risk (RH = 1.7, 95% CI 1.1–2.7).
Yuzbashian et al. (34)	Iran (Tehran)	PC	1,100/2,715/2,089	≥18 y; no CKD; hyperglycemia/ dyslipidemia/ hypertension	Low-sodium DASH (DASH score based on 168-item FFQ)	Median 3.12 y	High vs. low adherence linked to ∼40% ↓ CKD risk across high-risk groups.
Salomo et al. (36)	Denmark	RCT	18	≥18 y; CKD 3–4	NNRD vs. habitual diet	1 week each	NNRD vs. habitual: ① 24-h urinary P ↓313 mg/day; ② FEP ↓11%; ③ iP-FGF23 ↓30 pg/mL.
Hansen et al. (37)	Denmark	RCT	18	≥18 y; CKD 3–4	NNRD vs. habitual diet	1 week each	NNRD vs. habitual: ① NAE ↓80%; ② U-PCS ↓31%; ③ U-IS ↓29%
Hansen et al. (38)	Denmark	RCT	58	≥18 y; CKD 3–4	NNRD vs. habitual diet	26 weeks	NNRD vs. habitual: ① 24-h urinary P ↓19%; ② 24-h urinary Na ↓33%; ③ Proteinuria ↓39%; ④ Weight ↓2.2%.
Chang et al. (42)	Taiwan, China	CS	100	≥18 y; CKD 3–5	VD vs. omnivore (validated by 24-h recall)	N/A	VD vs. omnivore: serum phosphate significantly lower.
Hou et al. (46)	Taiwan, China	CS	2797	≥40 y; diabetes	VD vs. vegan vs. omnivore (FFQ)	N/A	CKD prevalence: VD 28.5%; vegan 30.4%; omnivore 36.3%.
Kuo et al. (47)	Taiwan, China	CS	6567	≥40 y; no CKD	VD vs. vegan vs. omnivore (FFQ)	N/A	CKD prevalence: VD 26.2%; vegan 32.7%; omnivore 30.7%.
Dinu et al. (49)	Italy	RCT	107	Adults; low-moderate CVD risk; no CKD	VD vs. MD (3-months periods, 24-h recall, MD score)	3 months each	VD period: SCr ↓5.3%; BUN ↓8.7%; eGFR ↑3.5%. MD groups: no significant change.
Shi et al. (56)	China	CS	8429	≥18 y	Traditional southern pattern vs. modern pattern (factor analysis)	N/A	Southern pattern Q4 vs. Q1: ↑ CKD odds (OR = 4.56, 95% CI 3.18–6.56). Modern pattern Q4 vs. Q1: ↓ CKD odds (OR = 0.50, 95% CI 0.36–0.71).

BUN, blood urea nitrogen; CKD, chronic kidney disease; CVD, cardiovascular disease; DASH, Dietary Approaches to Stop Hypertension; eGFR, estimated glomerular filtration rate; ESRD, end-stage renal disease; FEP, fractional excretion of phosphorus; FFQ, food frequency questionnaire; FGF23, fibroblast growth factor 23; HR, hazard ratio; iP-FGF23, intact plasma FGF23; IS, indoxyl sulfate; MD, Mediterranean Diet; NAE, Net Acid Excretion; NNRD, New Nordic Renal Diet; OR, odds ratio; P, phosphorus; PCS, p-cresyl sulfate; Q1/Q5, quintile 1/quintile 5; RH, relative hazard; SCr, serum creatinine; U-IS/PCS, urinary indoxyl sulfate/p-cresyl sulfate; VD, Lacto-Ovo Vegetarian Diet; y, years; ↑/↓, increase/decrease; RCT, randomized controlled trial; PC, prospective cohort; CS, cross-sectional.

### 2.4 Quality assessment study selection and characteristics

Two reviewers (HX and CY) independently assessed the risk of bias for the included studies. The revised Cochrane risk-of-bias tool for randomized trials (RoB 2) was used to assess randomized controlled trials (10). For observational studies (including cohort and cross-sectional studies), which aimed to evaluate the effect of dietary patterns as an exposure factor on outcomes, the Risk Of Bias In Non-randomized Studies - of Interventions (ROBINS-I) tool was employed (11). Any disagreements arising during the evaluation process were first discussed and resolved by two reviewers (HX and CY) through deliberation on the assessment criteria. If a consensus could not be reached after discussion, a third reviewer (PJ) was consulted to arbitrate and make the final decision.

### 2.5 Study election and characteristics

**FIGURE 2:**
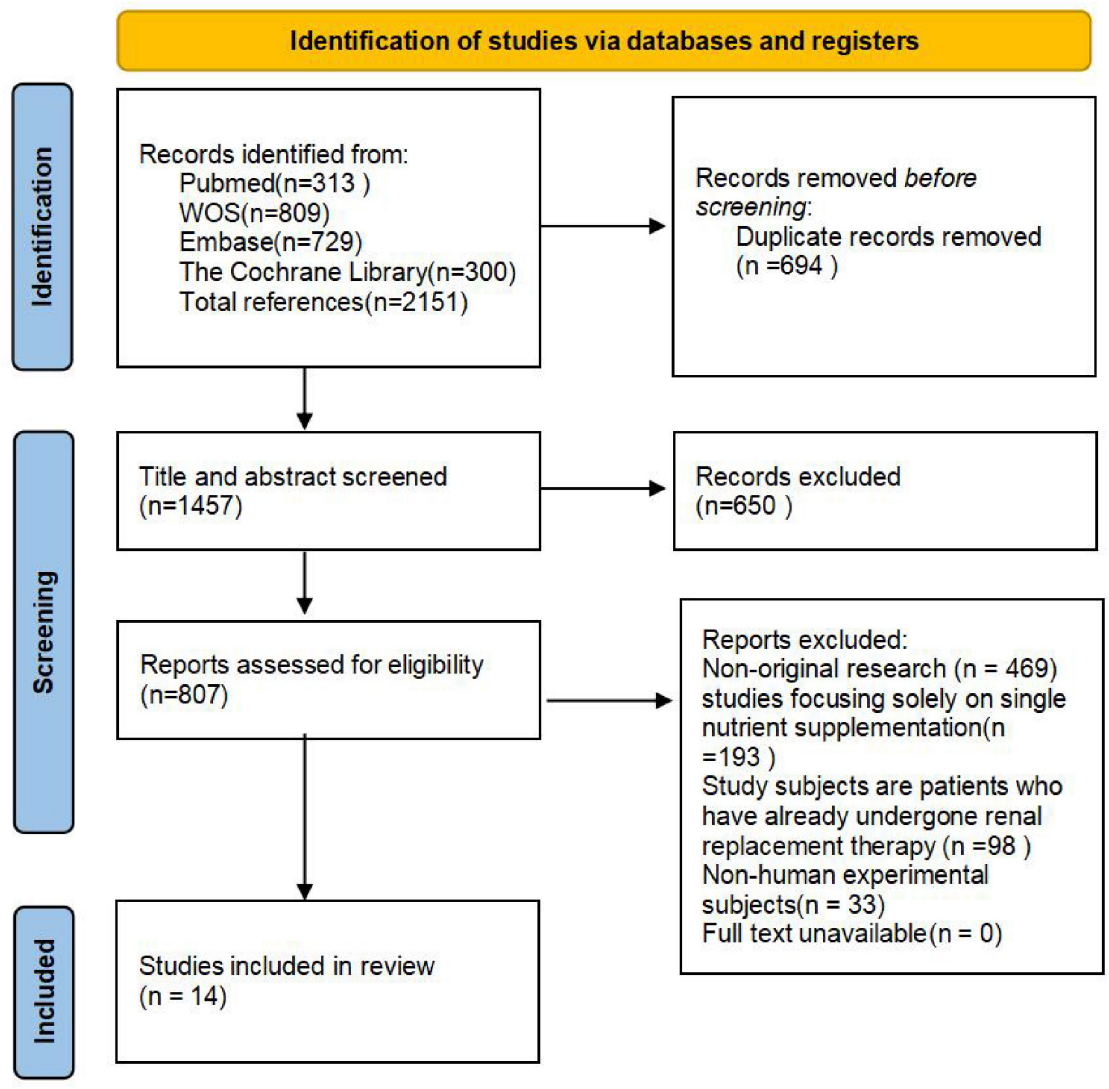
Preferred Reporting Items for Systematic Reviews and Meta-Analyses (PRISMA) flow diagram of the selection of the articles included.

As shown in Figure 2, the initial search yielded a total of 2151 records. After removing duplicates and screening titles and abstracts, 807 articles were retrieved for full-text assessment. Ultimately, 14 studiesmet the inclusion criteria and were included in this systematic review Table 1.

It should be noted that the studies included in this review exhibit heterogeneity in several key aspects, which should be considered when interpreting the overall conclusions. Firstly, different studies employed different dietary adherence scoring systems (e.g., various scoring scales were used to assess MD and DASH). Secondly, the study populations ranged from healthy community-dwelling adults to patients with different stages of CKD, with variations in baseline risk and renal function status. Finally, the definitions and measurement methods for outcomes such as CKD occurrence rate and progression differed across studies. Given the aforementioned heterogeneity, a narrative synthesis was adopted in this review to present the findings, thereby avoiding indiscriminate generalization of conclusions to all populations.

Among these 14 studies, four were randomized trials and ten were observational studies. Specifically, three studies investigated the effects of the MD on CKD, three studies analyzed the effects of the DASH on CKD, three studies evaluated the impact of the NNRD on renal function parameters in CKD patients, four studies examined the effects of the VD on CKD, and one study assessed the association between a Chinese dietary pattern and CKD.

**FIGURE 3:**
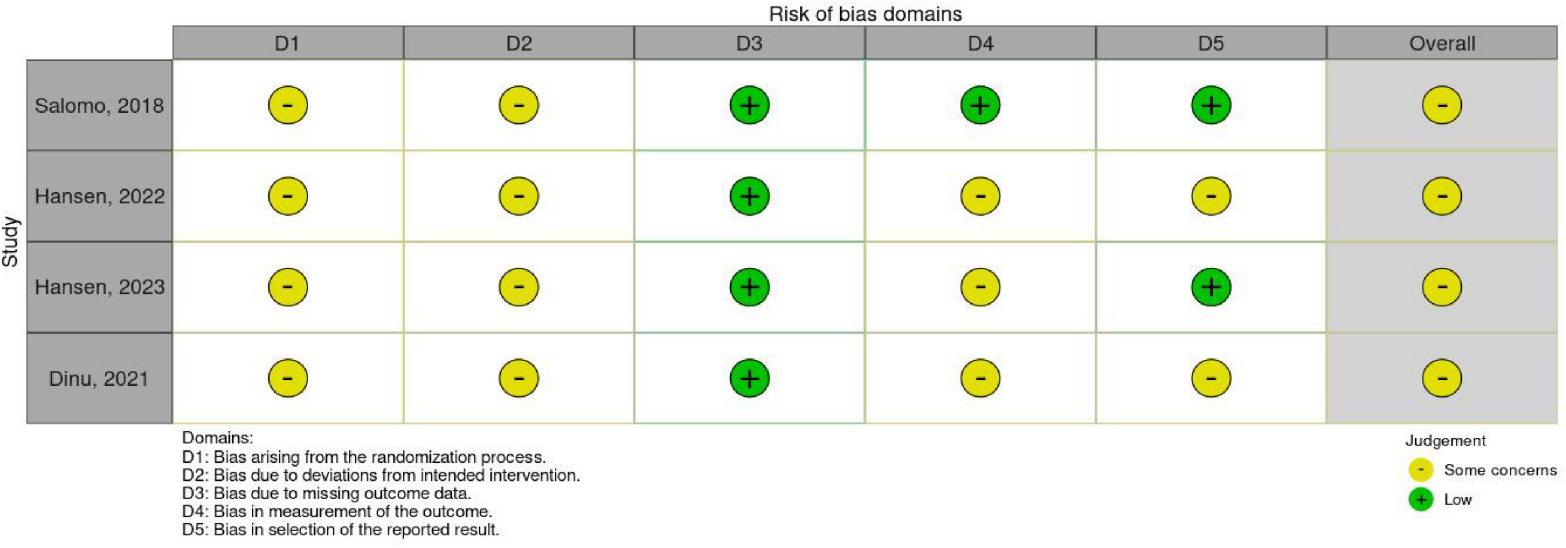
Methodological quality of the studies using the tool RoB 2.0.

**FIGURE 4:**
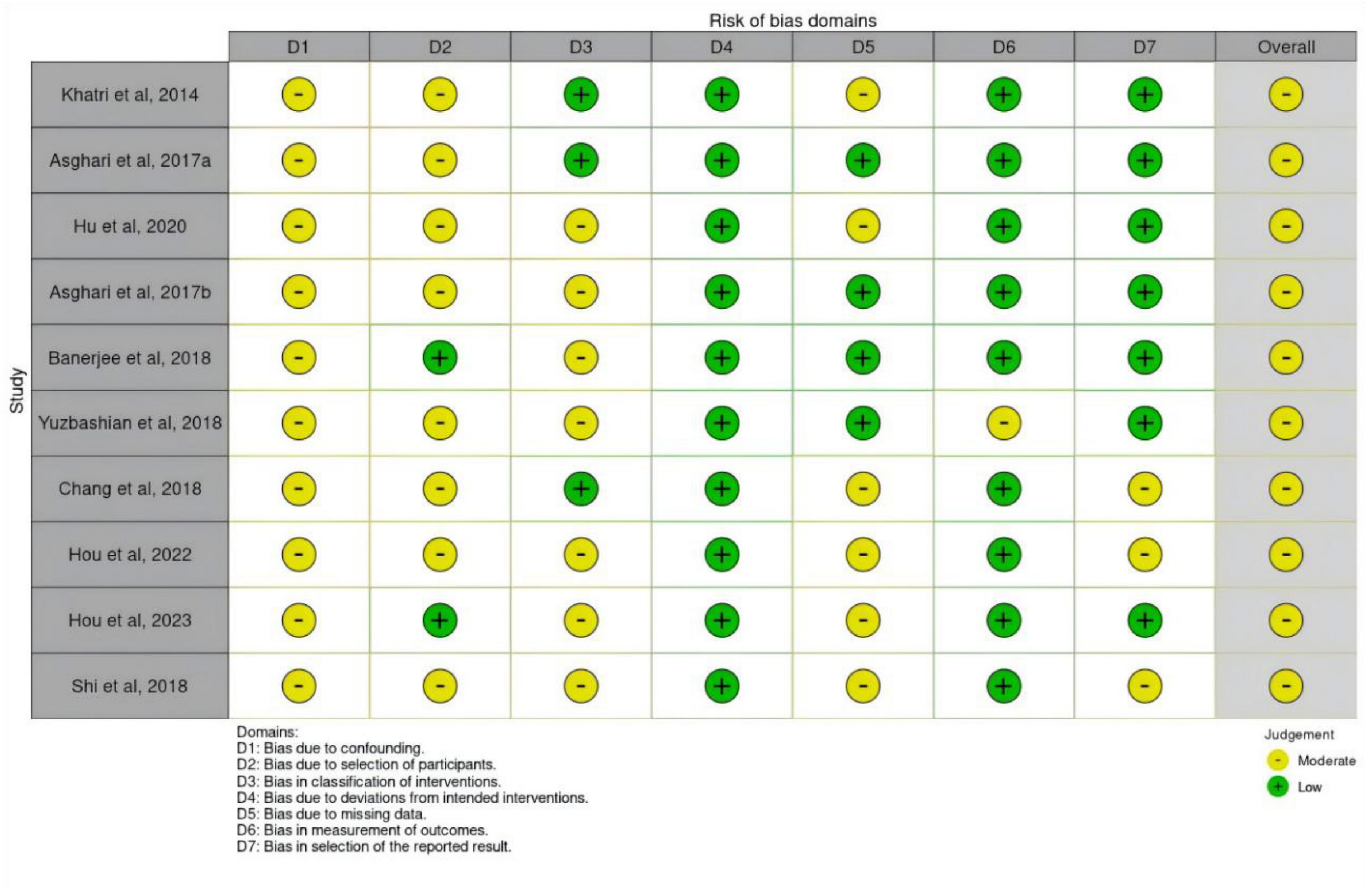
Methodological quality of the studies using the tool ROBINS-I.

Regarding methodological quality, according to the RoB 2 tool assessment, the overall risk of bias in the four randomized controlled trials (RCTs) was judged as “some concerns” Figure 3; whereas based on the ROBINS-I tool assessment, the overall risk of bias in the ten observational studies was judged as “moderate” Figure 4.

## 3 Mediterranean Diet (MD)

The concept of the “Mediterranean Diet” was first introduced by Keys and Grande in the early 1960s. It reflects a traditional dietary pattern observed in Greece and southern Italy, characterized by a high proportion of plant-based foods such as cereals, legumes, fresh fruits, green vegetables, nuts, and seeds. This diet also emphasizes adequate intake of fish and seafood, moderate consumption of dairy products, poultry, eggs, and alcohol (primarily wine consumed with meals), and the use of extra-virgin olive oil (cold-pressed) as the primary source of added fat. Red meat consumption is relatively low in this dietary pattern (12). Additionally, the MD emphasizes the significance of regular moderate physical activity and a short rest after lunch (13). The origins of MD can be traced back to a study covering seven countries (14, 15). This study found that residents in the Mediterranean region had a relatively low incidence of coronary heart disease (CHD), which might be closely related to their unique dietary habits. A recent study revealed that adherence to MD is associated with a reduced risk of CKD progression and its complications (8). Medical nutrition therapy (MNT) remains the cornerstone of CKD management, and an increasing number of researchers have shifted their focus to the multifaceted effects of MD on CKD.

In the NOMAS study, a community-based prospective cohort study in northern Manhattan (16), investigated the relationship between the MD and renal function. They tracked 900 participants aged 55 and older, who had no history of stroke or MRl contraindications, for 6.9 years. Throughout this period, they evaluated the participants’ Mediterranean diet (MeDi) scores and their correlation with eGFR to deduce the link between MD and the risk of CKD. The MeDi score spans from 0 to 9, with higher scores indicating greater adherence to MD. The study revealed that for each one-point increase in the MeDi score, there was a 17% decrease in the likelihood of having an eGFR < 60 ml/min per 1.73 m^2^. Moreover, participants with a MeDi score ≥ 5 had a 50% reduced risk of developing an eGFR < 60 ml/min per 1.73 m^2^ compared to those with a score <5. These results imply that following to MD may provide protective benefits for renal function, even in non-Mediterranean areas.

In a prospective study conducted in Tehran, Iran (17), followed 1,212 adults for 6 years. Dietary habits were assessed using a traditional Mediterranean diet score (MDS) based on food intake, ranging from 0 to 8. The median MDS for all participants was 4 (interquartile range: 3–5). The study found that the incidence of CKD was 19%. After adjusting for all possible confounding factors, individuals in the highest quartile of MDS exhibited a 51% lower risk of developing CKD compared to those in the lowest quartile.

The MD includes various beneficial components, such as fruits, vegetables, nuts, and whole grains, which are rich in antioxidants, fiber, and micronutrients including vitamins C and E, folate, magnesium, and potassium. These components are believed to prevent the decline in glomerular filtration rate (GFR), renal failure, and promote renal function (18, 19). Furthermore, the high consumption of fish rich in ω-3 fatty acids, as part of MD, can improve lipid levels, improve insulin resistance, endothelial function, and lower blood pressure, serum creatinine, and inflammatory markers (20, 21). These effects significantly contribute to preventing potential risk factors for CKD, such as obesity, diabetes, and cardiovascular diseases, offering theoretical support for the MD’s efficacy in reducing the risk of CKD.

Recent research suggests that the MD may help delay the progression of CKD. In the Chronic Renal Insufficiency Cohort (CRIC) study conducted in the United States (22), followed 2,403 participants with an eGFR between 20 and 70 ml/min/1.73 m^2^ for up to 14 years. The results indicated that participants who adhered most strictly to the alternate Mediterranean diet (aMed) had a 25% lower risk of CKD progression compared to those with the least adherence. The aMed refers to a dietary pattern modeled after MD but modified to suit specific conditions or needs (23). It highlights a high intake of plant-based foods, including plant proteins, monounsaturated fats, and fish, coupled with a reduced intake of products and saturated fats.

The MD, which emphasizes a high intake of fruits and vegetables, may raise concerns about hyperkalemia. However, a cross-sectional study conducted in the United States (24), found that a high potassium intake from plant-based diets was associated with a reduced risk of CKD, reflecting the overall beneficial effects of fruits and vegetables. Nevertheless, the CRIC study (22) demonstrated that higher urinary potassium excretion was associated with an increased risk of CKD progression, which contrasts with the generally assumed benefits of plant-based diets. This finding suggests a complex relationship between potassium intake and CKD progression, indicating that its specific effects may depend on the patient’s renal function status and other contextual factors.

## 4 Dietary Approaches to Stop Hypertension (DASH)

The Dietary Approaches to Stop Hypertension (DASH) was originally mentioned and developed in a large randomized controlled trial (NHLBI) initiated in the United States in 1994 (25). This study aimed to explore the effects of different dietary combinations on the treatment of hypertension. The DASH primarily consists of eight food groups: fruits, vegetables, nuts and legumes, whole grains, low-fat dairy products, sodium, red and processed meats, and sugar-sweetened beverages (26). Among these, it is particularly recommended to increase the intake of fruits, vegetables, nuts and legumes, whole grains, and low-fat dairy products while reducing the intake of sodium (<3 g/day), red and processed meats, and sugar-sweetened beverages. Given that hypertension is a strong and independent risk factor for CKD (27), many scholars have begun to explore the potential link between the DASH dietary pattern and CKD.

In a study conducted in Tehran, Iran (28), researchers using a validated and reliable 168-item food frequency questionnaire tracked subjects initially free from CKD for 6.1 years, assessing their adherence to the DASH. The results indicated that subjects in the top quintile of DASH adherence had an 18% lower risk of developing CKD compared to those in the bottom quintile. A systematic review and meta-analysis, based on data from prospective cohort studies, further supported that the DASH has a positive effect on preventing CKD (29). Concurrently, researchers observed that low adherence to DASH was associated with a higher risk of ESRD among adults in the third stage of CKD. In a study involving 1,110 adults (with an eGFR of 30–59 ml/min/1.73 m^2^), the percentage of ESRD events was 24.5% for those with the lowest quintile of DASH adherence scores (quintile 1), whereas it was 15.9% for those with the highest quintile (quintile 5) (30). Furthermore, research has also revealed that following the DASH in conjunction with a low-sodium diet can lower the risk of developing CKD among high-risk populations. The concept of a low-sodium DASH was initially introduced through the DASH-Sodium randomized crossover trial, which was designed by NHLBI in 1997. This trial categorized the sodium content in the DASH into three levels: 3, 6, and 9 g, corresponding to a low-sodium DASH, a standard DASH, and a DASH with sodium content akin that of to a typical American diet (26, 31, 32).

Subsequently, numerous countries and regions began to study modified sodium DASH. Various research results indicated that adhering to the DASH, combined with a low-sodium diet, could reduce risk factors for chronic non-communicable diseases, such as blood pressure, blood sugar, and blood lipids (33–35).

In the Tehran Lipid and Glucose Study (TLGS), a community-based prospective follow-up investigation conducted in Tehran, Iran (34), researchers tracked 1,100 subjects with abnormal blood glucose, 2,715 with abnormal blood lipids, and 2,089 with hypertension over 3 years. The study utilized a food frequency questionnaire (FFQ) to assess subjects’ intake of eight food groups and nutrients and calculated an overall score for a low-sodium DASH-style diet ranging from 8 to 40. None of the subjects had CKD at baseline. The 3-years follow-up results demonstrated that among subjects with dysglycemia, dyslipidemia, and hypertension, the occurrence rates of CKD in the high-adherence group to the low-sodium DASH were 16.3%, 15.1%, and 15.7%, respectively. Compared with their corresponding low-adherence groups, the risk of CKD incidence was reduced by approximately 40% in each case (34). This protective association remained statistically significant after adjustment for a range of potential confounding factors.

## 5 New Nordic Renal Diet (NNRD)

The New Nordic Renal Diet (NNRD) is an adaptation of the New Nordic Diet (NND), modified to exclude phosphorus-rich foods such as red bread, dairy products, nuts, and fish, while maintaining nutritional balance and limiting phosphorus intake to 850 mg per day. This dietary pattern originated from a randomized crossover trial study conducted in 2019 (36), which compared a week of habitual diet with a week of NNRD among 18 patients with CKD stages 3–4. The results indicated that this novel dietary approach reduced participants’ 24-h urinary phosphorus excretion by nearly 40%, decreased the urinary phosphorus excretion fraction by 29%, and lowered plasma intact FGF 23 levels by 18%. Since then, the NNRD has gradually gained attention.

In a randomized crossover trial conducted in Denmark (37), 18 patients with CKD stages 3–4 were subjected to a comparison between their habitual diet and the NNRD over a 1-week period. The focus was primarily on the effects of NNRD on uric acid excretion and uremic toxins. The findings revealed that, compared to the habitual diet group, patients who followed the NNRD for 7 days experienced a significant 80% reduction in 24-h net uric acid excretion. Additionally, due to the high levels of plant-based protein in the NNRD, there was a notable decrease in the 24-h urinary excretion of p-cresyl sulfate (PCS) and indoxyl sulfate (IS) by 31% and 29%, respectively, compared to the habitual diet group. In a subsequent 26-weeks randomized trial conducted in Denmark (38), 60 adults over the age of 18 with moderate CKD and an eGFR of 20–45 ml/min/1.73 m^2^ were compared while following the NNRD and their habitual diet. The objective was to investigate the renal effects of this novel dietary pattern, which is characterized by its low content of phosphorus, protein, and sodium. The findings revealed that, compared to the group on their habitual diet, the NNRD group experienced a 19% reduction in 24-h urinary phosphorus excretion, a 33% decrease in 24-h urinary sodium excretion, a 39% reduction in proteinuria, and an average weight loss amounting to 2% of their total body weight. Furthermore, the study examined the association between the NNRD and health-related quality of life (HRQoL) (39), revealing that the NNRD group experienced an overall improvement of 23% in their ability to perform daily activities during the intervention, which was correlated with a significant reduction in 24-h urinary phosphorus excretion. Similarly, a decrease of 26% in pain/discomfort during the intervention was associated with a substantial reduction in body fat mass. However, a study by Lee et al. (40) reported inconsistent findings. This study evaluated the effects of a low-protein diet (≤0.8 g/kg/day) on HRQoL outcomes in 571 patients with CKD and found that the low-protein diet was significantly associated with impaired HRQoL and depressive symptoms (40). These findings suggest that the impact of different protein restriction regimens on patient quality of life may vary, and their specific effects should be comprehensively evaluated in conjunction with the overall dietary composition and patient background.

## 6 Lacto-Ovo Vegetarian Diet (VD)

The Lacto-Ovo Vegetarian Diet (VD) is a dietary choice that focuses on plant-based foods, emphasizing a diverse intake of fruits, vegetables, whole grains, legumes, nuts, and seeds. This diet also permits the consumption of dairy products (such as milk, cheese, and yogurt) and eggs, while excluding meat, poultry, and seafood (41). In recent years, the application of the VD in the field of metabolic health and chronic disease management has gradually increased.

A cross-sectional study conducted in Taiwan analyzed 100 subjects and categorized them into two groups based on their dietary protein sources: a VD group and an omnivorous group. The study revealed that the serum phosphate level in the VD group was significantly lower than that in the omnivorous group (3.9 ± 0.7 mg/dl vs. 4.4 ± 1.5 mg/dl), indicating that this dietary pattern might help reduce the risk of hyperphosphatemia (42). Hyperphosphatemia is a prevalent issue among patients with pre-dialysis CKD, as the compromised kidneys cannot efficiently excrete phosphorus. This can lead to secondary hyperparathyroidism and elevated levels of fibroblast growth factor 23 (FGF-23), which, in turn, triggers increased urinary phosphorus excretion as a compensatory mechanism to maintain normal phosphorus balance (43). Therefore, the pathological process in which the aforementioned compensatory mechanisms may ultimately decompensate suggests that proactive intervention and control of serum phosphorus levels in the early stages of the disease hold profound potential value for preventing clinical deterioration in patients with CKD.

Furthermore, previous studies have indicated that the bioavailability of phosphorus in plant-based diets (approximately 50%) is lower than that in animal-based diets (approximately 70%). This difference is primarily due to the phytates abundant in plant-based foods, which can bind to phosphorus and inhibit its absorption (44). Studies by Moe et al. have also confirmed that compared to meat-eaters, patients on a vegetarian diet for 1 week exhibited lower serum phosphorus levels, decreased FGF-23 levels, and increased urinary phosphorus excretion fractions (45).

In a retrospective cohort study conducted by Hou et al. in Taipei (46), the dietary habits of 2,797 adults aged 40 and above were analyzed through a questionnaire survey. Participants were categorized into vegan, VD, and omnivore groups. Utilizing a structural equation model for analysis, the results indicated that the VD group had a lower probability (28.5%) of developing CKD compared to the omnivore group (36.3%) and the vegan group (30.4%). The study further stratified subjects with an HbA1c level greater than 6.5% or a history of diabetes mellitus over 40 years old. Through modeling, it was discovered that a Lacto-Ovo Vegetarian Diet had a direct effect on reducing the incidence of CKD among diabetic patients, potentially linked to increased insulin sensitivity associated with this dietary pattern (41).

In a similar study (47), the research team examined dietary habits among 6,567 adults aged 40 and older, dividing participants into vegan, lacto-ovo vegetarian, and omnivore groups. They discovered that the lacto-ovo vegetarian group had a lower probability of CKD (26.2%) compared to both the omnivore group (30.7%) and the vegan group (32.7%), and also exhibited a reduced incidence of moderate-to-severe non-alcoholic fatty liver disease (NAFLD) (32.9%). This incidence was significantly lower than that of the vegan group (35.6%) and the omnivore group (40%). These findings are consistent with those reported by Nazra (48), indicating that VD may offer therapeutic benefits for NAFLD and positively influence overall health and the reduction of chronic disease risk.

Chronic kidney disease is an independent risk factor for cardiovascular disease. In a randomized, open-label, crossover trial, researchers assessed the impact of a VD and a MD on renal function among 107 subjects with low-to-moderate cardiovascular risk profiles. In this study, 54 participants adhered to the VD, while 53 followed the MD. Both dietary interventions lasted 3 months. It was observed that during the intervention period, the VD group experienced a 5.3% decrease in serum creatinine (Scr), an 8.7% decrease in blood urea nitrogen (BUN), and a 3.5% increase in eGFR. In contrast, no significant changes in the aforementioned parameters were observed in the Mediterranean diet group (49). This study preliminarily suggests a positive effect of the lacto-ovo-vegetarian diet on short-term renal function improvement. However, the generalizability of these effects across diverse populations and their long-term sustainability should be key focuses of future research.

## 7 Chinese dietary patterns

Since the reform and opening up, the sustained and rapid growth of the Chinese economy has driven significant changes in the dietary structure of Chinese adults. Specifically, there has been a decline in the intake of grains and vegetables, while the consumption of animal-based foods, primarily pork, has increased. Meanwhile, the intake of eggs, fish, and dairy products remains at a relatively low level, and the consumption of cooking oil and salt far exceeds the recommended standards, leading to a high proportion of fat energy intake (50). However, due to China’s vast territory and significant differences in dietary culture and habits across regions, a uniformly defined dietary pattern has not yet emerged. Instead, there is a coexistence of multiple dietary patterns with local characteristics.

In recent years, the relatively low incidence of CKD in the southeastern coastal regions of China has garnered scholarly attention (51). Researchers hypothesize that this phenomenon may be closely linked to the unique dietary habits of the locale. Drawing from the dietary culture and customs of the southeast coastal areas, three healthy dietary patterns have emerged: the “Oriental Healthy Dietary Pattern,” the “Lingnan Dietary Pattern,” and the “Jiangnan Dietary Pattern” (52–54). All three patterns advocate a staple diet of grains, complemented by a rich variety of vegetables, fruits, soy products, dairy products, and seafood, while emphasizing cooking methods that use less oil and salt.

Specifically, the Oriental Healthy Dietary Pattern emphasizes grains as the staple food, accompanied by an abundance of vegetables, fruits, dairy products, soy products, and seafood, with a recommendation for low-oil and low-salt cooking method (52). The Lingnan Dietary Pattern, similar to the former, also focuses on a high intake of vegetables and fruits, as well as moderate amounts of meat, eggs, and dairy, incorporating the unique dietary culture of the Lingnan region with an emphasis on “frequent soup cooking and a focus on nourishment” (53). The Jiangnan Dietary Pattern, on the other hand, centers on fresh vegetables, fruits, whole grains, soy products, and freshwater fish, advocating limited intake of red meat and high-sugar foods, with an overall dietary style that is bland (54).

Furthermore, the Chinese Nutrition Society has also introduced the “Balanced Dietary Pattern for Chinese Residents,” aiming to provide more scientific dietary guidance for the population. This pattern suggests increasing the intake of grains, vegetables, and fruits, consuming moderate amounts of animal products, soybeans, and nuts, while reducing the use of oil and salt, and advocating for adequate water intake and active physical activities (55). Although these dietary patterns are believed to have a positive effect on reducing the incidence of CKD, there is currently no direct research clarifying their specific impact on CKD. Further studies are needed to explore the specific role of these dietary patterns in the prevention and treatment of CKD.

A study based on the China Health and Nutrition Survey (CHNS) analyzed the association between dietary patterns and CKD among the general population in China. The final sample included 8429 participants, and individual dietary intake data were collected using a 24-h dietary recall method for three consecutive days (56). Dietary patterns were constructed using factor analysis, and the results showed that a traditional southern dietary pattern characterized by high intake of rice, pork, and vegetables and low intake of wheat was associated with a more than 4.5-fold increase in the prevalence of CKD. The researchers speculated that this association might be related to the distribution of cadmium in the diet, as cadmium is mainly found in rice, vegetables, wheat, and pork (57). Conversely, the study found that a modern dietary pattern (high intake of fruits, soy milk, eggs, milk, fried foods, fast food, and pastries) was negatively associated with the prevalence of CKD. However, the average fat and protein intakes increased with higher quartiles of this pattern, while carbohydrate intake decreased. Notably, higher scores for the modern dietary pattern were generally associated with a higher average BMI among participants (56). Previous studies have shown that the modern dietary pattern is positively correlated with obesity among older adults in China (58), and obesity has been proven to be associated with an increased risk of CKD (59). This suggests that although the modern dietary pattern is associated with a lower prevalence of CKD, its high-fat and high-protein characteristics may indirectly have negative impacts on kidney health by increasing the risk of obesity.

The complex findings from these observational studies not only reveal the unique challenges of exploring the diet-CKD relationship in the Chinese population but also point to the deeper reasons for the continued scarcity of high-level evidence for CKD prevention and management. This situation primarily stems from the following challenges: first, there are limitations in research methodology. Existing studies rely heavily on cross-sectional studies or interventions targeting intermediate CKD risk factors (e.g., hypertension, cardiovascular disease), while more advanced study designs capable of establishing causality (e.g., prospective cohort studies, randomized controlled trials) remain relatively scarce. Second, there is a gap in the complete evidence chain. Current evidence focuses predominantly on demonstrating improvements in risk factors, whereas the longitudinal evidence chain translating these effects to CKD incidence and progression (e.g., decline in eGFR, ESRD) has not yet been fully established. Furthermore, Chinese society is undergoing a unique nutritional transition. The coexistence of traditional and Westernized dietary patterns has resulted in a complex dietary landscape and introduced significant confounding factors, making it particularly difficult to accurately assess the independent effect of any single dietary pattern on CKD. Therefore, directly equating any Chinese dietary pattern with a well-established CKD intervention strategy should be avoided. A more objective approach, based on current research, is to view them as exploratory frameworks for constructing future localized strategies. The potential value and current limitations of this framework are clearly exemplified in the research on the “Chinese Heart-Healthy Dietary Pattern” (CHH).

To evaluate the impact of CHH on blood pressure and CVD risk, a multicenter, randomized, double-blind, parallel-controlled trial was conducted in four cities in China: Beijing, Shanghai, Guangzhou, and Chengdu, representing the four major culinary traditions of Lu, Huaiyang, Cantonese, and Sichuan, respectively (60). This study enrolled 265 Chinese adults aged 25–75 years with a baseline systolic blood pressure (SBP) of 130–159 mmHg. Participants were randomly assigned to the CHH group or the regular diet group for a 28-days intervention. The results showed that the CHH group experienced an average reduction of 10.0 mmHg in SBP and 3.8 mmHg in diastolic blood pressure (DBP). Additionally, at the end of the intervention, the estimated 10-years CVD risk was significantly lower in CHH group, with a relative change of −37.4%, compared to −10.4% in the regular diet group. These findings confirm the beneficial effects of CHH on key risk factors for CKD (hypertension and cardiovascular disease), while its renoprotective efficacy must be ultimately verified through rigorously designed, long-term, kidney outcome-driven trials.

## 8 Discussion

This study systematically reviewed epidemiological evidence regarding the associations between the MD, DASH, NNRD, VD and Chinese dietary patterns with CKD Table 2. The analysis indicates that although these dietary patterns differ significantly in food composition and cultural origins, they all exert positive effects on both the primary prevention and management of CKD through distinct mechanisms. However, the applicability of these patterns in the context of Chinese dietary habits, target populations, and strength of evidence varies considerably. Thus, their effectiveness and feasibility require further clarification and validation through localized studies.

**TABLE 2:** The comparison between among different dietary.

Dietary components	MD	DASH	NNRD	VD	Chinese balanced diet
Fruits	1–2 Svs/meal	4–5 x/d	Berries 50–100 g/d+ Apples/pears 100 g/d	1–2 Svs/d	200–350 g/d
Vegetables	≥2 Svs/meal	4–5 x/d	Root veg 200–250 g/d+ Leafy greens 100–150 g/d	5∼6 Svs/d	300∼500 g/d
Grains	1–2 Svs/meal (preferably whole grains)	7∼8 x/d	Whole grains (rye/oats) 150–200 g/d	2∼3 Svs/d	Grains 200–300 g/d (whole grains and legumes 50–150 g/d) Tubers 50–100 g/d
Dairy and dairy products	2 Svs/d	2∼3 x/d (low-fat or fat-free)	Low-fat milk/oat milk 100–150 ml/d	4∼5 Svs/d	300∼500 g/d
Legumes	≥2 Svs/w	Nuts, seeds, and legumes 4–5 x/w	30–50 g/d	1∼2 Svs/d	Soy and nuts 25–35 g/d
Olive, nuts, and seeds	1∼2 Svs/d		Almonds/hazelnuts 10–15 g/d (unsalted)	2∼3 Svs/d	
Meat and meat products	Red meat<2 Svs/w White meat 2 Svs/w Processed meat ≤ 1 Svs/w	Lean meat, poultry, and fish ≤ 2 x/d	Red meat ≤ 1 x/w; Avoid processed meats	/	Animal-derived foods 120–200/d (seafood ≥ 2 x/d, 1 egg/d)
Fish/seafood	≥2 Svs/w		3∼4 Svs/w	/	
Eggs	2∼4 Svs/w		3∼4 Svs/w	3∼4 Svs/d	
Fats, oils	/	Fats, oils 2∼3 x/d	Canola oil/flaxseed oil 15–20 ml/d	/	Oils 25–30 g/d
Sweets	≤2 Svs/w	≤5 x/w	≤5 x/w	/	/
Others	Potatoes ≤ Svs/w Regular physical activity Adequate rest	Salt < 2300 mg/d	Salt ≤ 2000 mg Phosphorus ≤ 800 mg	/ /	Water 1500∼1 700 ml/d Salt < 5 g 6000 steps/d
Effects on chronic kidney disease	Preventing the decline of GFR, slowing the progression of CKD	Preventing the decline of GFR, slowing the progression of CKD	Slowing the decline of kidney function, improving metabolic indicators	Preventing the decline of GFR, slowing the progression of CKD	It remains unclear
Evidence sources	Cohort study, cross-sectional study, systematic review	Cohort study, RCT, systematic review	RCT	Cross-sectional study, cohort study, systematic review	Guidelines/expert consensus

Svs/meal, servings per meal; Svs/d, servings per day; Svs/w, servings per week; x/d, times per day; x/w, times per week; veg, vegetables; CKD, chronic kidney disease; GFR, glomerular filtration rate; RCT, randomized controlled trial.

### 8.1 Common foundations and distinct pathways

First, a horizontal comparison of the mechanisms of action of different dietary patterns revealed that all included patterns share the core characteristic of a high intake of plant-based foods (e.g., fruits, vegetables, whole grains). The antioxidants, anti-inflammatory compounds, and dietary fiber abundant in these foods collectively constitute a potential common foundation for delaying the progression of CKD. Building upon this commonality, different patterns exhibit their distinct features. As the most extensively studied representatives, the benefits of the MD and DASH patterns primarily stem from the effective control of traditional risk factors for CKD, such as hypertension and cardiovascular disease. The MD may confer broad indirect renal protection through synergistic effects of its key components–such as unsaturated fatty acids and polyphenols from extra virgin olive oil, and ω-3 fatty acids from fish–which exert anti-inflammatory effects, improve lipid metabolism and insulin resistance, and protect endothelial function (18–21). Concurrently, the habitual moderate consumption of red wine between meals in the MD is considered a potential protective factor; its active components (e.g., hydroxytyrosol) have demonstrated antioxidant and anti-fibrotic activities in experimental studies (12, 61). Furthermore, the MD advocates for regular moderate physical activity, which has also been associated with a lower occurrence rate of CKD and delayed progression (62). Overall, these interrelated biological effects–from anti-inflammatory and antioxidant actions to improvements in metabolic and endothelial function–collectively form the potential physiological basis by which the MD delays CKD progression.

The core mechanism of the DASH pattern lies in its systematic adjustment of electrolytes and dietary structure, emphasizing “low sodium, high potassium, high magnesium,” and the intake of low-fat dairy products (26). Its protective effects are likely mediated through multiple pathways: sodium restriction directly reduces volume load (63); adequate potassium and magnesium may synergistically regulate blood pressure by promoting urinary sodium excretion and improving vascular reactivity, among other mechanisms (64–66). The calcium, potassium, and bioactive proteins (e.g., whey protein) in the low-fat dairy products advocated by DASH are also hypothesized to improve insulin sensitivity and reduce oxidative stress, thereby protecting vascular endothelial function (67). Through the systematic control of hypertension–a central driver of CKD progression–the above mechanisms collectively constitute the physiological basis by which DASH delays renal function decline.

In contrast, NNRD and VD demonstrate more direct interventions targeting the core pathological processes of CKD. As exemplars of precision nutrition, NNRD is specifically designed to address three major challenges in advanced-stage CKD: protein-energy wasting, phosphorus/uric acid retention, and uremic toxin accumulation. Through systematic restriction of dietary phosphorus (≤850 mg/day) and total protein, with an emphasis on plant-based proteins and low-phosphorus dairy products, NNRD not only reduces exogenous phosphorus and nitrogen waste load but has also been shown to effectively lower serum FGF-23 levels and fractional excretion of urinary phosphorus (36, 37, 39). Furthermore, its high dietary fiber content is believed to modulate gut microbiota, potentially suppressing the production of gut-derived uremic toxins (such as indoxyl sulfate and p-cresyl sulfate) and promoting their fecal excretion, thereby establishing a “secondary detoxification pathway” independent of renal function (36).

Lacto-Ovo Vegetarian Diet, on the other hand, precisely regulates mineral metabolism through a distinct mechanism, primarily attributed to the significantly lower bioavailability of plant-derived phosphorus (approximately 30%–50%) compared to animal-derived phosphorus (approximately 70%) (44). This implies that even with equivalent phosphorus intake, vegetarians absorb and circulate less phosphorus systemically, thereby helping prevent hyperphosphatemia at the source. Effective control of serum phosphorus levels alleviates persistent stimulation of the FGF-23–klotho axis, which may subsequently delay the progression of secondary hyperparathyroidism (68). Additionally, VD may offer multi-faceted protection in CKD management through synergistic mechanisms such as correction of metabolic acidosis, modulation of the gut–kidney axis via dietary fiber to reduce uremic toxins, and utilization of phytochemicals to counteract oxidative stress and inflammation (49, 69).

These mechanistic distinctions suggest that in clinical practice, MD and DASH are more suitable for primary prevention in the general population and early-stage CKD management, whereas NNRD and VD provide more targeted tools for precision nutrition intervention in patients with advanced CKD.

However, compared to the aforementioned dietary patterns with well-established international consensus, research on the Chinese dietary pattern remains in the exploratory and definitional stage. Rooted in local dietary culture, it may possess inherent advantages in terms of cultural adaptability and long-term adherence. Currently, there is no single unified definition; instead, multiple healthy paradigms have emerged, represented by the “Oriental Healthy Dietary Pattern” and “Jiangnan Dietary Pattern,” among others. These patterns generally emphasize rice or whole grains as staple foods, abundant intake of vegetables, fruits, soy products, and freshwater fish and shrimp, along with strict control of oil and salt consumption (52–55). Structurally, it is inferred that such patterns may reduce the risk of major CKD risk factors, such as hypertension and diabetes, through mechanisms similar to those of the MD and DASH–namely, high antioxidant content, high fiber, and low saturated fat–thereby potentially offering indirect protection for renal function. Nevertheless, their specific effects and underlying mechanisms require direct validation through local studies.

### 8.2 A critical examination of geographical applicability

Although the aforementioned international dietary patterns demonstrate potential in the prevention and management of CKD, their applicability within the context of China’s unique dietary culture and epidemiological background must be systematically evaluated as a critical issue. This evaluation encompasses three dimensions: cultural compatibility, food accessibility, and evidence generalizability.

Firstly, the core food components of dietary patterns originating from specific regional cultures may differ significantly from traditional Chinese dietary habits. For instance, the central roles of extra virgin olive oil, specific fish types, and red wine in the MD (12), and the reliance on Nordic-specific berries and rye bread in the NNRD (36), exhibit profound differences from the inherent dietary habits and structures of the general Chinese population, making deep integration difficult. Forced promotion could lead to poor adherence and even induce new nutritional issues due to inappropriate substitutions. Secondly, while the DASH and VD are beneficial in principle, the popularity, economic cost, and role within the daily culinary system of certain specific components they advocate (e.g., low-fat dairy products, specific nuts, or whey protein) in China challenge their feasibility for large-scale implementation.

Most critically, there are limitations regarding the generalizability of the evidence. The majority of current research demonstrating the benefits of MD, DASH, NNRD, and VD has been conducted in Western or specific regional populations (e.g., Denmark, Iran). Differences in genetic background, gut microbiota, baseline disease spectrum, and lifestyle among different populations create uncertainty when directly extrapolating these findings to the Chinese population. For example, while the high intake of potassium-rich fruits and vegetables in the MD is beneficial for Western populations with normal renal function, it may pose a potential risk of hyperkalemia for China’s vast population of advanced CKD patients, who often have low awareness rates of their condition. Similarly, the efficacy demonstrated by the NNRD in Danish patients with moderate CKD may not be replicable in Chinese patients with distinct metabolic characteristics and dietary backgrounds.

This critical examination does not negate the value of these patterns but aims to emphasize that a simple “adoptionism” approach is inadvisable. Instead, it highlights the urgency and necessity of developing healthy dietary patterns that are based on China’s native dietary culture, targeted toward the epidemiological characteristics of the Chinese population, and validated by local clinical trials in China. Utilizing international evidence as a theoretical reference constitutes a more scientific research pathway.

### 8.3 Methodological considerations and risk of bias

Through a critical appraisal of the included study designs, methodological limitations and potential risks of bias that may affect the robustness of the conclusions were identified. First, the body of evidence in this review is primarily derived from observational studies, with residual confounding–particularly “healthy user bias”–representing a major source of potential bias. Although most studies adjusted for a range of known confounders, individuals adhering to healthy dietary patterns tend to exhibit more favorable lifestyle behaviors and greater health awareness, which are themselves associated with a lower risk of CKD. Consequently, the observed protective associations may partly reflect the influence of such unmeasured or residual confounding, potentially leading to biased estimation of the true effect of dietary patterns.

Secondly, although these studies provide a higher level of evidence for causal inference, they are generally limited by small sample sizes (*n* = 18–107) and short intervention durations (1–26 weeks), which restricts the generalizability of their findings to long-term clinical endpoints such as end-stage renal disease. Furthermore, due to the specific nature of dietary interventions, blinding of participants and intervention implementers is practically challenging, potentially leading to performance bias and compromising the objectivity of the results.

In summary, these biases and limitations imply that the results of observational studies may overestimate the true protective effects of dietary patterns, whereas findings from experimental studies primarily confirm their short-term efficacy in improving surrogate endpoints such as biomarkers. The absolute long-term benefits of these dietary patterns on CKD outcomes still require validation through larger-scale studies with extended follow-up. Nonetheless, the consistent trends observed across different study designs and populations provide a degree of support for the core findings of this review.

## 9 Conclusion

In conclusion, current evidence indicates that the MD and DASH are currently the most compelling dietary patterns for the prevention of CKD. The NNRD and VD offer valuable alternatives for specific CKD populations, such as those with hyperphosphatemia or metabolic abnormalities. However, the extrapolation of all international dietary patterns to the Chinese population requires careful consideration. A more fundamental issue is the lack of high-level research evidence supporting a healthy dietary pattern that aligns with the dietary habits of Chinese residents and leverages the advantages of local foods.

## 10 Prospects

To achieve the goal of establishing a dietary prevention and management system for CKD in China, future research should follow a phased and systematic approach. In the short term, priority should be given to conducting adaptive intervention trials based on the principles of the MD or DASH in Chinese populations. These trials should aim to evaluate the feasibility, adherence, and preliminary efficacy of these dietary patterns within the context of Chinese dietary habits. Building upon this foundation, medium-term research should initiate large-scale prospective cohorts focusing on indigenous dietary patterns, such as the “Jiangnan Diet” and “Lingnan Diet,” to systematically observe their long-term associations with CKD incidence and progression. This will provide high-level epidemiological evidence for the selection of optimal dietary models. Ultimately, the long-term objective is to design and implement multicenter, randomized controlled dietary intervention trials targeting Chinese CKD patients or high-risk populations. The goal is to obtain conclusive local efficacy evidence, thereby laying a solid scientific foundation for establishing CKD prevention and management dietary guidelines with distinctive Chinese characteristics.

## 11 Strengths and limitations

This study’s strength lies in its systematic integration and comparison of five major dietary patterns currently associated with CKD–namely, the MD, DASH, NNRD, VD and the Chinese dietary pattern. It is also the first review to incorporate the emerging NNRD, offering a contemporary perspective in this field. Furthermore, beyond mere description, this study preliminarily compares the functional characteristics of different dietary patterns. For instance, it highlights that MD and DASH primarily focus on controlling CKD risk factors, whereas NNRD and VD place greater emphasis on directly managing CKD complications such as hyperphosphatemia. This comparison provides an initial framework for understanding the clinical application contexts of different dietary patterns. Finally, this study critically examines “regional applicability” as a core issue and systematically outlines the current status of the Chinese dietary pattern, offering targeted suggestions for future localized research.

However, this study also has several limitations. The primary limitation is that most of the included original studies are observational (e.g., cohort and cross-sectional studies) or small-sample randomized controlled trials, which offer limited levels of evidence and make it difficult to establish a clear causal relationship between dietary patterns and CKD outcomes. Additionally, there were variations in the dietary assessment methods used across the original studies, such as differences in dietary adherence scoring, which may affect the direct comparability of the results. Moreover, although the search strategy aimed to be comprehensive, it may still have missed some relevant studies due to database coverage and language restrictions, introducing the possibility of publication bias.
